# AI in Aesthetic/Cosmetic Dermatology: Current and Future

**DOI:** 10.1111/jocd.16640

**Published:** 2024-11-07

**Authors:** Sukruthi Thunga, Marius Khan, Soo Ick Cho, Jung Im Na, Jane Yoo

**Affiliations:** ^1^ New York University New York New York USA; ^2^ Avisé Labs GmbH Dortmund Germany; ^3^ Lunit Seoul Republic of Korea; ^4^ Department of Dermatology, Seoul National University Bundang Hospital Seoul National University Seoul Republic of Korea; ^5^ Mount Sinai School of Medicine New York New York USA

**Keywords:** aesthetic, cosmetic dermatology, dermatology, digital imaging

## Abstract

**Background:**

Recent advancements in artificial intelligence (AI) have significantly impacted dermatology, particularly in diagnosing skin diseases. However, aesthetic dermatology faces unique challenges due to subjective evaluations and the lack of standardized assessment methods.

**Aims:**

This review aims to explore the current state of AI in dermatology, evaluate its application in diagnosing skin conditions, and discuss the limitations of traditional evaluation methods in aesthetic dermatology. Additionally, the review proposes strategies for future integration of AI to address existing challenges.

**Methods:**

A comprehensive review of AI applications in dermatology was conducted, in both diagnostic and aesthetic fields. Traditional methods such as subjective surveys and hardware devices were analyzed and compared with emerging AI technologies. The limitations of current AI models were evaluated, and the need for standardized evaluation methods and diverse datasets was identified.

**Results:**

AI has shown great potential in diagnosing skin diseases, particularly skin cancer. However, in aesthetic dermatology, traditional methods remain subjective and lack standardization, therefore limiting their effectiveness. Emerging AI applications in this field show promise, but they have significant limitations due to biased datasets and inconsistent evaluation methods.

**Conclusions:**

To develop the potential of AI in aesthetic dermatology, it is crucial to create standardized evaluation methods, collect diverse datasets reflecting various ethnicities and ages, and educate practitioners on AI's utility and limitations. Addressing these challenges will improve diagnostic accuracy, better patient outcomes, and help integrate AI effectively into clinical practice.

## 1 Introduction

### 1.1 The Current Implementation of Artificial Intelligence (AI) in Dermatology

Recent advancements in AI have significantly transformed the field of dermatology, particularly in detecting and diagnosing skin diseases. A landmark study in Nature demonstrated deep neural networks' efficacy in classifying skin cancer, achieving performance comparable to dermatologists [1]. This highlights AI's potential to provide diagnostic support and extend dermatologists' reach beyond traditional settings [1].

AI models are increasingly used as diagnostic support tools in dermatology by leveraging image analysis, especially in primary care settings or by non‐specialists [2]. Recent studies highlight AI‐based algorithms for skin cancer detection in mobile health (mHealth) apps, making this technology accessible to the public [3, 4, 5]. These methods aid in the early detection of skin diseases, such as cancer and infectious skin diseases, potentially reducing morbidity, mortality, and transmission rates [6, 7].

Beyond skin disease diagnosis, AI has shown promise in various dermatological applications, such as assessing the severity of atopic dermatitis, psoriasis, and alopecia areata [8, 9, 10]. AI's ability to analyze large datasets and provide real‐time support makes it a critical tool for the future of dermatology, enhancing healthcare providers' capabilities and improving patient outcomes.

Aesthetic dermatology, focused on improving skin appearance, faces challenges distinct from medical dermatology. While medical dermatology diagnoses are based on diagnostic criteria or confirmed by biopsy, defining conditions like wrinkles or pigmentation is less clear in cosmetic dermatology. Medical dermatology also uses validated severity scales like the Eczema Area and Severity Index (EASI), Psoriasis Area Severity Index (PASI), and Severity of Alopecia Tool (SALT). In contrast, aesthetic evaluations are subjective, lacking validated tools and typically involve concerns like wrinkles, pigmentation, and skin laxity, which vary based on age, race, and ethnicity [11, 12].

AI has the potential to revolutionize aesthetic dermatology by offering more consistent and objective evaluations [13]. AI algorithms can analyze large datasets, identifying patterns and correlations not evident through human observation, enhancing accuracy and monitoring changes over time. However, AI's application in this field is still in its early stages. This comprehensive review explores the current state of AI in aesthetic dermatology, evaluates traditional methods' limitations, examines AI's emerging role, and discusses the challenges and opportunities for AI integration into clinical practice.

## 2 The Current State of Assessment in Aesthetic/Cosmetic Dermatology and Its Limitations

Aesthetic dermatology has recently advanced in treating conditions such as wrinkles, pigmentation, and skin laxity due to innovations in related technologies. Various methods have been proposed for assessing cosmetic skin conditions, including subject questionnaires, instrumental measurements, and overall assessments by evaluators [12, 14, 15].

One common method for assessing skin conditions through questionnaires is the Baumann Skin Type Indicator, which uses standardized questions about hydration, sensitivity, pigmentation, and wrinkles to classify skin into 16 distinct types. While this method allows for systematic categorization at low cost, it is limited by the subjectivity of responses and its binary classification, which fails to reflect detailed conditions. Device measurements, typically conducted with tools like the Mexameter, Sebumeter, Cutometer, and Tewameter, offer objective and precise data but often focus on localized rather than global skin assessments [15]. Overall assessments by evaluators, though comprehensive, tend to be subjective and coarsely graded, making subtle changes harder to detect.

Additionally, no universal standards exist for assessing specific cosmetic conditions, unlike the EASI for atopic dermatitis or the PASI for psoriasis [16]. A further challenge is the diverse aging patterns targeted by aesthetic dermatology, including wrinkles, pigmentation, and sagging skin, which vary significantly across ethnicities. Current measurement methods are not well‐equipped to handle these variances efficiently [17].

This lack of standardized evaluation methods leads to several limitations. First, it diminishes the ability to compare the effectiveness of different treatments in aesthetic dermatology, creating a barrier to advancing evidence‐based medicine by either encouraging more effective treatments or discouraging less effective ones. Second, the difficulty in accurately assessing changes before and after treatment limits precision medicine, which aims to identify the best approach for each patient. Lastly, the lack of standardized measurement methods makes it challenging to collect large‐scale data on aesthetic/cosmetic dermatology.

## 3 Imaging Hardware Devices and AI Algorithms

Recent advances in camera imaging equipment have led to the introduction of dedicated hardware devices for evaluating facial skin conditions, now commonly utilized in clinics for aesthetic dermatology assessments. These devices collect multimodal data, which can be combined with other patient information to train machine‐learning algorithms, enhancing the precision of skin assessments.

### 3.1 Canfield

Canfield offers advanced imaging devices like the Vectra H2, IntelliStudio, Visia Skin Analysis, Vectra WB360, Vectra XT, and Reveal Imager, utilizing RBX technology to separate red and brown skin components, and gray mode to reveal contours. They feature markerless tracking for skin surface assessments, simulate volume changes, and visualize contours with a color distance map. Canfield devices measure spots, wrinkles, texture, and pores, and simulate 3D results for adding or removing volume. Though they provide high‐resolution 2D and 3D imaging, their clinical superiority over conventional methods remains unproven, and high costs may limit accessibility, potentially exacerbating healthcare disparities.

### 3.2 Quantificare

Quantificare offers imaging solutions like the LifeViz Mini Face, LifeViz Infinity Face, Body & Breast, LifeViz Body, and LifeViz Micro Skin Microstructure. These devices visualize facial shapes from any angle, assess facial measurements, and compare them to the golden ratio. They provide detailed analyses of wrinkle depth, pore size, oiliness, vascularization, pigmentation, and can quantify volume changes post‐treatment, as well as evaluate skin tightening and lifting in 3D. However, the impact of Quantificare's 3D data on clinical decision‐making is not yet fully established, requiring further research on patient outcomes.

### 3.3 Miravex

Miravex's Antera 3D device uses a patented method to reconstruct 3D images of the skin surface, tagged to a 3D mannequin for detailed analysis of wrinkles, texture, pores, and volume. This tool provides a comprehensive understanding of skin conditions and aids in planning and monitoring aesthetic treatments. However, its effectiveness across diverse skin types and impact on clinical outcomes require further investigation. Comparative studies with other imaging systems would help clinicians select the most suitable technology for their practices (Table 1).

**TABLE 1:** Examples of AI technologies in aesthetic dermatology.

Company	Device	Software capabilities	Measurements (face)	AI capabilities
Canfield	Vectra H2, IntelliStudio, Visia Skin Analysis, Vectra WB360, Vectra XT, Reveal Imager	RBX technology, gray mode, markerless tracking	Spots, wrinkles, texture, pores, brown spots, red areas	Simulate 3D results, overlay preoperative images with simulated outcomes
Quantificare	LifeViz Mini Face, LifeViz Infinity Face Body & Breast, LifeViz Body, LifeViz Micro Skin Microstructure, LifeViz Mini Pro for Face	Visualization of facial shapes, analysis of wrinkle depth, pore size, oiliness, vascularization, pigmentation	Facial angles, measurements, height, widths	Automatic image organization
Miravex	Antera 3D	Reconstruction of full 3D images of the skin surface	Wrinkles, skin texture, pores, volume	Detailed analysis and monitoring of aesthetic treatments
FotoFinder	ATBM Master, Meesma, Trichoscale DX	Automated lesion detection, PASI scoring, high‐resolution imaging	Lesion detection, hair and scalp analysis	Automated analysis and documentation of skin conditions
Aram Huvis	AI‐scalp grader	Automated hair and scalp analysis	Scalp condition assessment	Assessment based on scalp photographic index

### 3.4 Fotofinder

FotoFinder offers imaging solutions like the ATBM Master for comprehensive skin analysis, automated lesion detection, and Meesma for high‐resolution handheld imaging. These devices visualize skin conditions, texture, and pigmentation, and monitor changes over time. The ATBM Master also provides automated PASI scoring for large areas [18].

The Moleanalyzer pro, FotoFinder's AI‐based tool for skin lesion assessment, was validated in studies like “Man against machine” and “Man against machine reloaded” at Heidelberg University [19]. In the “Man against machine” study, a deep learning CNN for melanoma recognition achieved an AUROC of 0.86, comparable to 58 dermatologists [20]. The Moleanalyzer pro uses a modified version of Google's Inception v4, trained on dermoscopic images from the ISIC archive, and validated with external databases like Memorial Sloan Kettering and the ISIC‐2018 challenge datasets [21].

FotoFinder also offers Trichoscale DX for automated hair and scalp analysis, providing data for both long and clipped hair. While AI enhances diagnostics, concerns may arise about over‐reliance on technology in clinical decision‐making.

### 3.5 Aram Huvis

The AI‐scalp grader is an automated hair and scalp analysis device using 60‐fold magnification images captured by a trichoscope (ASM‐224S, Aram Huvis Co. Ltd.). It assesses scalp condition based on the Scalp Photographic Index, classifying scalp types and scoring five features: dryness, oiliness, erythema, folliculitis, and dandruff [22]. In a clinical study, scalp cosmetics prescribed based on the AI‐scalp grader's assessment improved scalp conditions [23]. However, its real‐world impact on patient outcomes in trichology and dermatology requires further study. Its focus on specific scalp features may limit applicability across all hair types and colors, necessitating broader evaluation in diverse populations.

### 3.6 User Experiences and Clinical Feedback

Clinical reports highlight both the advantages and challenges of these imaging systems. Users report improved patient communication and more precise treatment planning. Canfield's VISIA is praised for detailed skin analyses, while Quantificare's LifeViz is valued for volume analysis in filler treatments. However, a steep learning curve, data privacy concerns, and high acquisition costs are common challenges.

AI tools like FotoFinder's Moleanalyzer pro receive positive feedback, especially for early skin cancer detection, though many physicians stress it should complement, not replace, clinical expertise. Despite the benefits of advanced imaging and AI in dermatology, their real‐world impact requires further scrutiny. Systems like Canfield, Quantificare, and FotoFinder provide high‐resolution imaging and detailed analyses, but clinical outcomes remain underexplored. The integration of AI raises ethical concerns regarding data privacy and over‐reliance on technology. Comparative studies are needed to guide technology selection, and high costs may limit accessibility, requiring ongoing evaluation of long‐term benefits across diverse skin types. Balancing innovation with ethical considerations will be key as the field progresses.

## 4 Applying Multimodal Data—Combining Sensor Data With Additional Metadata of the Patient

The integration of multimodal data, combining various data types, is crucial for training machine learning algorithms, particularly in the medical field. In dermatology, using multimodal data in AI applications has shown significant promise. For example, machine learning algorithms have been applied to analyze dermoscopic images instrumental in diagnosing and grading a patient's lesion condition. This approach leverages their rich visual information ito improve diagnostic accuracy and consistency.

Moreover, integrating clinical images and metadata from smartphones has been used to develop deep‐learning models for skin lesion classification [24]. By combining images with patient metadata, such as demographics and lesion characteristics, these models can provide more accurate, personalized diagnoses. Studies show that incorporating patient information like age, gender, and medical history enhances AI performance [25, 26]. Dermatologists consider these factors when making diagnostic judgments, and AI models that do the same are more likely to replicate the nuanced decision‐making processes of human experts.

Research highlights the importance of integrating diverse data sources to improve the accuracy and reliability of dermatological diagnoses [27]. Combining skin sensor data with patient metadata offers a more comprehensive understanding of an individual's skin condition. Devices like Courage + Khazaka's Multi Probe Adapter System MPA and Corneometer CM 825, along with others, measure skin parameters like moisture, sebum, elasticity, and pigmentation. When combined with patient metadata and clinical data, this multimodal information can enhance machine learning algorithm training. This approach is beneficial in aesthetic/cosmetic dermatology, where treatments must be customized to individual skin characteristics and preferences.

While the potential benefits of multimodal data integration are clear, several technical and practical challenges remain. One major hurdle is data compatibility, as imaging devices and sensors often use different formats, making it difficult to standardize and share data across platforms. This lack of uniformity creates barriers to seamless integration and can result in inconsistent or incomplete datasets. Without standardized protocols, the reliability and generalizability of AI models may be compromised.

Additionally, robust data governance frameworks are needed to protect patient privacy, particularly with sensitive data like facial images. Developing clear data governance policies—such as anonymization techniques and secure storage protocols—is essential to safeguard patient data and foster trust in AI systems.

Despite these challenges, successful multimodal data integration is emerging. Recent oncology studies, for instance, have demonstrated the efficacy of combining histopathological, radiological, and genomic data to improve risk stratification and treatment outcomes [28, 29]. These examples highlight AI's potential to leverage diverse data sources to enhance diagnostic accuracy and clinical decision‐making, offering valuable insights for dermatology.

The integration of multimodal data not only enhances AI diagnostic capabilities but also supports precision medicine. By providing a comprehensive view of the patient's skin health, AI can identify subtle patterns and correlations that might be missed by traditional methods, leading to more effective, individualized treatments.

## 5 Applying AI in Aesthetic/Cosmetic Dermatology

### 5.1 Attempts to Apply AI in Aesthetic/Cosmetic Dermatology and Their Limitations

The application of artificial intelligence (AI) in aesthetic and cosmetic dermatology is an emerging field that holds great potential for improving patient care and treatment outcomes. Despite advancements in aesthetic/cosmetic dermatology involving the use of advanced imaging systems and software algorithms, several challenges and limitations must be addressed to fully realize AI's potential in this field.

One major limitation is the lack of comprehensive datasets that reflect diversity in the patient population. Most AI models are trained on datasets that may not adequately represent different ages, skin types, and ethnicities, leading to potential biases and less accurate diagnoses for certain groups. A 2023 study emphasized the complexity of creating representative datasets for various skin types and ethnic groups, highlighting the underrepresentation of minorities in clinical studies and significant differences in skin barrier function, such as a higher number of corneocyte layers in Black individuals. This underscores the need for AI systems that account for diversity [30].

Another challenge is the variability of input data. Imaging devices and sensors can vary in their measurements, causing measurement inconsistencies that affect AI performance. The subjective nature of some assessment methods, such as evaluator assessments, introduces further variability, limiting these models' effectiveness. Despite these challenges, AI‐based systems are gaining importance. A 2023 study found that AI algorithms in smartphone apps performed comparably to experts in diagnosing pigmented skin lesions, though doctors were superior in making treatment decisions [31].

In addition to technical limitations, integrating AI into clinical practice requires addressing ethical and regulatory concerns, particularly around patient privacy and data security. Since aesthetic/cosmetic dermatology often involves sensitive personal information, it is crucial that AI models follow strict ethical guidelines to protect patient confidentiality and prevent data misuse. A 2024 study demonstrated that using explainable AI (XAI) in a skin cancer diagnostic system improved AI acceptance by providing explanations for AI decisions, which increased dermatologists' trust in the system and their own diagnoses [32].

While AI offers significant opportunities for advancing aesthetic/cosmetic dermatology, addressing these limitations—through standardized methods, diverse datasets, and ethical practices—will be key for successful AI integration and achieving more accurate, personalized, and effective treatments for patients.

### 5.2 Suggestions for Applying AI in Aesthetic/Cosmetic Dermatology

In aesthetic dermatology, the ability to comprehensively quantify a patient's skin aesthetic issues using AI, combined with hardware device measurements and clinician assessments, will be a major catalyst for advances. To achieve this, several prerequisites must be met.

First, standardization in aesthetic evaluation is essential. Unifying units of measurement and establishing standards across different devices is necessary. Developing aesthetic/cosmetic metrics that encompass various ages and ethnicities is crucial. Research on correlations between assessment methods, such as comparing inter‐device measurements and subjective evaluations to objective device measurements, will provide an important basis for determining standardized methods [33]. Standardization will facilitate consistent, reliable assessments, enhancing the credibility and utility of AI in dermatology.

Second, a wide range of data must be collected through standardization. Datasets should reflect diverse ages and ethnicities, including results from multiple instruments or evaluators. Ethnic diversity is crucial as aging varies by ethnicity [17]. Building these diverse datasets will help develop AI models that incorporate various aspects, ensuring they are not biased toward specific races, ages, or equipment [34, 35].

Third, making data and AI models as public as possible will drive progress. Open sharing of data and models has historically accelerated AI advancements [36]. Public data in aesthetic dermatology will help AI models perform well in various environments. However, since dermatology data often includes facial images, posing privacy risks, models that protect data confidentiality may be needed. Generated data through generative models can offer a solution [37, 38, 39].

Fourth, education and guidance for practitioners and clients are necessary to ensure effective AI use in aesthetic dermatology. Understanding AI's utility and limitations in real‐world applications will help users maximize its benefits. Conversely, AI developers must thoroughly understand aesthetic dermatology to create effective solutions. Educating practitioners on AI's capabilities and limitations will promote informed decision‐making and better integration of AI into clinical practice.

International cooperation is crucial to building these prerequisites. Recently, several guidelines for skin AI research have been published [40, 41]. Similarly, a working group of dermatologists, industry stakeholders, and regulators is needed to create guidelines for AI applications in this field. This group should oversee standardization, research, product development, and real‐world AI implementation. This cooperation will pave the way for seamless AI application in aesthetic/cosmetic dermatology, enhancing its value for patients and providers.

## 6 Conclusion

The advancement of imaging systems and AI technologies offers significant opportunities to enhance clinical practice in aesthetic/cosmetic dermatology. Realizing AI's potential requires the establishment of standardized evaluation methods, collection of diverse datasets representing various ethnicities and ages, and practitioner education on AI's applications and limitations. Addressing these challenges will enable successful AI integration into practice, improving diagnostic accuracy and patient outcomes.

## Author Contributions

All authors have made significant contributions to this work. All authors conceptualized and drafted the manuscript and contributed to the literature review and critical revisions. Jung Im Na and Jane Yoo provided essential insights and final approval of the manuscript. All authors have read and approved the final version of the manuscript.

## Ethics Statement

The authors have nothing to report.

## Conflicts of Interest

The authors declare no conflicts of interest.

## Data Availability

Data sharing is not applicable to this article as no new data were created or analyzed in this study.
